# Association of mannose-binding lectin-2 genotype and serum levels with prognosis of sepsis

**DOI:** 10.1186/cc8157

**Published:** 2009-11-05

**Authors:** Jin Won Huh, Kyuyoung Song, Jung-Sun Yum, Sang-Bum Hong, Chae-Man Lim, Younsuck Koh

**Affiliations:** 1 Department of Pulmonary and Critical Care Medicine, Inje University Ilsan Paik Hospital, 2240 Daehwa-dong, Goyang-si, 411-706, Korea; 2 Department of Biochemistry and Molecular Biology, University of Ulsan College of Medicine, 388-1 Pungnap-dong, Seoul, 138-736, Korea; 3 Dobeel Corporation, Byoksan Techonopia 407, 434-6 Sandaewon-dong, Seongnam-si, 462-716, Korea; 4 Department of Pulmonary and Critical Care Medicine, Asan Medical Center, University of Ulsan College of Medicine, 388-1 Pungnap-dong, Seoul, 138-736, Korea

## Abstract

**Introduction:**

Individuals deficient in mannose-binding lectin (MBL), an important component of the innate immune system, show increased susceptibility to infection. We investigated whether polymorphisms in the MBL2 gene and the serum level are associated with the severity and prognosis of sepsis.

**Methods:**

A total of 266 patients with sepsis and 398 healthy controls were enrolled. We analyzed the three single nucleotide polymorphisms (Gly54Asp, -550, and +4) in the MBL2 gene. Serum samples collected on day 1 were analyzed for the levels of MBL.

**Results:**

Patients who were heterozygous (A/B) or homozygous (B/B) at codon 54 (adjusted odds ratio (OR), 0.370; 95% confidence interval (CI), 0.207-0.661, *P *= 0.001) and who were heterozygous (H/L) or homozygous (L/L) at -550 (adjusted OR, 0.476; 95% CI, 0.249-0.910, *P *= 0.025) were less likely to have septic shock in the sepsis group. Using Cox regression analysis for 28-day mortality, an MBL level ≥ 1.3 microg/mL showed significantly lower 28-day mortality (*P *= 0.020; hazard ratio, 0.571; 95% CI, 0.355-0.916) in the septic shock group.

**Conclusions:**

Homozygosity at codons 54 (A/A) and -550 (H/H) appears to be associated with the severity, but not the outcome, of sepsis, whereas a low MBL level may be an independent risk factor for mortality. These findings suggest that the genotype and serum level for MBL2 may have different clinical implications.

## Introduction

Severe sepsis and septic shock cause 30% to 50% of all deaths in intensive care units (ICUs) [1]. Numerous studies have suggested that individuals vary in their ability to resist infection [2-4]. Genetic variations, such as those in the *TNF-α *alleles, have been implicated in determining the susceptibility to and outcome of sepsis [3,5-8]. The innate immune system is activated prior to the acquired immune system, and is thus the first line of defense against pathogens. The importance of the interactions between pathogen-associated microbial patterns and mannose-binding lectin (MBL) in activating innate immunity has been considered as a component of the innate immune system [9]. Moreover, it is now recognized that the first response to invasion (i.e., innate immunity) has a significant influence on the subsequent adaptive response [10,11].

MBL is a calcium-dependent collagenous lectin present in serum. The high-molecular-weight oligomeric form of MBL binds carbohydrates on the surface of bacteria, fungi, and parasites. MBL then mediates activation of the complement cascade through MBL-associated serine proteases (MASP)-1 and -2, resulting in the destruction of microorganisms by opsonization and direct complement-mediated death [12-14].

It has been reported that low concentrations of MBL cause defects in opsonization and phagocytosis that have been associated with recurrent infections in both infants and adults [15-17]. Low serum levels of MBL have been correlated with polymorphisms in the protein-coding region of *MBL*2 at codons 52, 54, and 57, which encode the variant alleles D, B, and C, respectively [18-20]. It was previously reported that two *MBL2 *polymorphisms (MBL-2 exon 1 and promoter -221) were associated with the development of sepsis, severe sepsis, and septic shock in Caucasian adults [21]. However, ethnic differences have been reported for both the promoter and structural variants, and large inter-individual variations in the level of MBL can be explained by the promoter variants [22]. Among Koreans, no polymorphisms in codons 52 and 57 have been reported, whereas polymorphisms in *MBL*2 at codons 54, -550 (promoter), and +4 (5'-UTR) have been associated with low MBL levels [23].

In this study, we investigated the relation between polymorphisms in *MBL*2 and the serum concentration of MBL, and assessed whether these polymorphisms influence the severity and prognosis of sepsis in a Korean population.

## Materials and methods

### Study population

Two hundred and sixty-six patients receiving intensive care for sepsis between 1 May, 2004 and 31 December, 2006 were enrolled in this study. All patients were managed according to our sepsis management protocol, which was guided by three full-time critical care physicians. All patients were older than 16 years of age (mean age ± standard deviation, 61.6 ± 14.7 years; male:female (M:F) = 169:97) and had been admitted to the ICU of a university-affiliated hospital in Seoul, Korea. The patients were divided into two groups: the severe sepsis group (mean age 61.6 ± 16.9 years; M:F = 45:32) and the septic shock group (mean age 61.6 ± 13.8 years; M:F = 124:65). The diagnosis of severe sepsis or septic shock was based on the criteria presented at the American College of Chest Physicians/Society of Critical Care Medicine Consensus Conference in 1992 [see Additional data file 1] [24]. As control subjects, 398 healthy blood donors (mean age 37.2 ± 14.2 years; M:F = 219:179) were recruited. Informed consent was obtained from all study participants in accordance with the policies of the Institutional Review Board. This study was approved by the Institutional Review Board of the Asan Medical Center, Seoul, Korea.

Clinical data, including demographic details, the Sequential Organ Failure Assessment (SOFA) score, the Acute Physiology, Age, and Chronic Health Evaluation II (APACHE II) score obtained at day one of severe sepsis or septic shock, and the ICU outcome, were recorded for each patient. Blood samples for MBL polymorphism and serum level were drawn within 24 hours of the onset of severe sepsis or septic shock

### Single nucleotide polymorphism genotyping

We chose three single nucleotide polymorphism (SNPs; -550 in the promoter, +4 in the upstream region, and Gly54Asp in the coding region), which had previously exhibited an association with low MBL levels [23]. Genotyping was performed by PCR and sequencing, as previously described [25]. Haplotype analysis (*A/B *at codon 54, *H/L *at -550, and *P/Q *at +4) was performed to characterize the combined effects of the polymorphisms [26].

The polymorphism of -550 in the promoter was amplified by PCR in a 302 bp fragment: forward primer 5'-TTGCCAGTGGTTTTTGACTC-3' and reverse primer 5'-GTATCTGGGCAGCTGATTCC-3'. The two polymorphisms of +4 in the upstream region and Gly54Asp were amplified by PCR in a 386 bp fragment: forward primer 5'-AGTCACGCAGTGTCACAAGG-3' and reverse primer 5'-AGAACAGCCCAACACGTACC-3'.

### Quantification of MBL by double antibody sandwich ELISA

The serum MBL level was measured using a sandwich ELISA (MBL-ELISA; Dobeel, Gyeonggi, Korea) according to a previously established protocol [23].

### Statistical analysis

The descriptive results of the continuous variables were expressed as medians with an interquartile range (IQR). All categorical data were compared using chi-squared analysis or a Fisher's exact test. Continuous data were compared using the Kruskal-Wallis or Mann-Whitney U tests. A receiver operating characteristic (ROC) curve was used to evaluate the cut-off values for the MBL level. A multiple stepwise logistic regression model was used to evaluate the prognostic value of the MBL level. The genotype frequencies were checked for consistency among cases and controls separately with those expected from the Hardy-Weinberg equilibrium [see Additional data file 2] using commercial software (SNP Alyze v 5.0; Dynacom, Yokohama, Japan). The association between cases and controls were examined by comparing allele and genotype frequencies in different groups of subjects using a chi-squared test. Allelic frequencies were compared between cases and controls using logistic regression to calculate age, gender-adjusted odds ratios (OR), and 95% confidence intervals (CI). Logistic regression analysis was also conducted to examine any significant association between polymorphism and disease phenotype (disease site and behavior). The pairwise linkage disequilibrium (LD) values, D', *R*^2^, and *P *values corresponding to chi-squared tests were calculated using the SNP Alyze software package (SNP Alyze v 5.0; Dynacom, Yokohama, Japan). The same software was used to estimate haplotypes and their frequencies. SNP Alyze software uses an expectation-maximization algorithm that determines the maximum-likelihood frequencies of multi-locus haplotypes in diploid populations. To examine differences in individual haplotype frequency and overall haplotype profiles between cases and controls, a permutation test was performed using the SNP Alyze software. In addition, *P *values were calculated by chi-squared statistics derived from simple two by two tables based on the frequency of each haplotype versus all others combined between cases and controls.

## Results

### Demographics of the subjects

The characteristics of the patients at the time of admission are shown in Table 1. The overall mortality rate at 28 days was 31.4%. The severe sepsis group had a lower SOFA score and lower mortality rate compared with the septic shock group (10.6% vs. 39.7%; *P *< 0.001).

**Table 1:** Baseline characteristics of the patients at day one of severe sepsis or septic shock

Characteristic	Severe sepsis (n = 77)	Septic shock (n = 189)	*P *value
Age	65 (57-71)	65 (53-71)	NS
Male gender, %	58.4	65.6	NS
APACHE II score	19 (15-24)	26 (21-34)	0.000
SOFA score	9 (7-11)	13 (10-15)	0.000
Admission route, n (%)			NS
Medical	73 (94.8)	167 (88.4)	
Surgical			
elective/emergent	3 (3.9)/1 (1.3)	18 (9.5)/4 (2.1)	
Prior or preexisting disease, n (%)			NS
Chronic liver disease	6 (7.8)	17 (9.0)	
Chronic pulmonary disease	1 (1.3)	7 (3.7)	
Congestive heart disease	2 (2.6)	4 (2.1)	
Diabetes mellitus		5 (2.6)	
Malignancy	14 (18.2)	44 (23.3)	
Neurologic disease	5 (6.5)	10 (5.3)	
Others^a^	3 (3.9)	7 (3.7)	
≥ 2 diseases	7 (9.1)	22 (11.6)	
None	39 (50.7)	73 (38.6)	
Type of infection, n (%)			0.034
Pneumonia	20 (26.0)	80 (42.3)	
Intraabdominal infection	15 (19.5)	34 (18.0)	
Biliary	20 (26.0)	19 (10.1)	
Urinary tract infection	11 (14.3)	21 (11.1)	
Bacteremia	2 (2.6)	4 (2.1)	
Wound infection	5(6.5)	12 (6.3)	
Others^b^	4 (5.2)	19 (10)	
Positive culture, n (%)	45.5	37.0	NS
Gram-negative	68.6	60.0	
Gram-positive	17.1	31.4	
Mixed	11.4	2.9	
Anaerobe	2.9	2.9	
Fungi		2.9	
Mechanical ventilation, %	51.9	79.3	<0.0001
Renal replacement, %	9.1	31.2	<0.0001
Length of ICU stay, days	4 (3-9)	10 (6-19)	<0.0001
Nosocomial infection, %	23.4	22.2	NS

Data are presented as the median and interquartile range (25% to 75%). ^a ^= rheumatologic disease, inflammatory bowel disease; ^b ^= cellulitis, meningitis, leptospirosis.

APACHE II = Acute Physiology, Age, and Chronic Health Evaluation II; ICU = intensive care unit; NS = not significant; SOFA = Sequential Organ Failure Assessment.

### The association of the MBL2 gene polymorphisms with sepsis susceptibility

Patients who were heterozygous (*A/B*) or homozygous (*B/B*) for the polymorphism at codon 54 (adjusted OR, 0.370; 95% CI, 0.207 to 0.661, *P *= 0.001) were less likely to have septic shock in the sepsis group (Table 2). Those patients in the sepsis group who were heterozygous (*H/L*) or homozygous (*L/L*) at -550 (adjusted OR, 0.476; 95% CI, 0.249 to 0.910, *P *= 0.025) were less likely to have septic shock (Table 3). The frequencies of *P/Q *at +4 were not significantly different among the three groups (data not shown).

**Table 2:** Genotype frequencies for Gly54Asp in mannose-binding lectin between patients and controls and between septic patients

Locus	Allele	Group N (%)		Group n (%)		OR (95% CI)	*P*	Adjusted OR^a^ (95% CI)	*P*
		Sepsis		Control					
GG	A	185	72.27	262	66.16				
GA		64	25.00	115	29.04	0.788 (0.550, 1.129)	0.194	0.815 (0.507, 1.312)	0.400
AA	B	7	2.73	19	4.80	0.522 (0.215, 1.266)	0.150	0.388 (0.127, 1.180)	0.095
GG		185	72.27	262	66.16				
GA/AA		71	27.73	134	33.84	0.750 (0.532, 1.058)	0.102	0.742 (0.471, 1.167)	0.196
		Septic shock		Severe sepsis					
GG	A	144	77.84	41	57.75				
GA		37	20.00	27	38.03	0.362 (0.199, 0.658)	0.001	0.368 (0.202, 0.672)	0.001
AA	B	4	2.16	3	4.23	0.389 (0.084, 1.807)	0.228	0.381 (0.081, 1.794)	0.222
GG		144	77.84%	41	57.75%				
GA/AA		41	22.16%	30	42.25%	0.365 (0.205, 0.650)	0.001	0.370 (0.207, 0.661)	0.001

^a ^= The adjusted OR was adjusted for age and gender. CI = confidence interval; OR = odds ratio.

**Table 3:** Genotype frequencies for --550 in mannose-binding lectin between patients and controls and between septic patients

Locus	Allele	Group n (%)		Group n (%)		OR (95% CI)	*P*	Adjusted OR^a^ (95% CI)	*P*
		Sepsis		Control					
GG	H	77	30.20	124	31.16				
GC		122	47.84	182	45.73	1.079 (0.749, 1.556)	0.682	1.060 (0.783, 3.598)	0.810
CC	L	56	21.96	92	23.12	0.980 (0.633, 1.518)	0.929	1.333 (0.755, 2.355)	0.322
GG		77	30.20	124	31.16				
GC/CC		178	69.80	274	68.84	1.046 (0.744, 1.472)	0.795	1.142 (0.734, 1.788)	0.555
		Septic shock		Severe sepsis					
GG	H	63	34.24	14	19.72				
GC		85	46.20	37	52.11	0.479 (0.239, 0.957)	0.037	0.465 (0.231, 0.936)	0.032
CC	L	36	19.57	20	28.17	0.381 (0.173, 0.840)	0.017	0.379 (0.171, 0.839)	0.017
GG		63	34.24	14	19.72				
GC/CC		121	65.76	57	80.28	0.484 (0.254, 0.922)	0.027	0.476 (0.249, 0.910)	0.025

^a ^= The adjusted OR was adjusted for age and gender. CI = confidence interval; OR = odds ratio.

### The association of serum MBL levels with the MBL2 genotypes

The distribution of MBL concentrations was closely associated with the various *MBL2 *genotypes. The *HH, HL*, and *LL *genotypes of the -550 polymorphism and the *AA, AB*, and *BB *genotypes of the codon 54 polymorphism were correlated with high, medium, and low MBL levels, respectively, in all three groups, whereas the *QQ, PQ*, and *PP *genotypes of the +4 polymorphism were correlated with high, medium, and low MBL levels, respectively, only in the control group (Table 4). The serum MBL level was different among the three groups, even for subjects with the same genotype. Among the subjects with genotype *HL/LL *at -550 and *PP *at +4, the serum MBL level was higher for those in the septic shock group than for those in the severe sepsis group (Table 4).

**Table 4:** Correlation of SNPs in the *mbl2 *gene with the serum MBL level

			Normal control		Severe sepsis		Septic shock	
			
Loci	Allele	Genotype	Median (IQR) μg/L	*P* ^a^	Median (IQR) μg/L	*P* ^a^	Median (IQR) μg/L	*P* ^a^
-550	H	GG	2493 (1452--3992)	0.000	2250 (1285--4800)	0.000	2550 (2010--3945)	0.000
		GC	955 (335--2626)		810 (600--1360)		1530^bc^ (860--2400)	
	L	CC	384 (0--1284)		340 (275--710)		640^c^ (300--1630)	
Gly54 Asp	A	GG	2249 (1333--3352)	0.000	1370 (825--3925)	0.000	2280 (1500--2800)	0.000
		GA	315 (3--540)		460^b^ (335--695)		475^b^ (298--695)	
	B	AA	0		270 (135-280)		270	
+4	P	CC	1039 (215--2668)	0.036	800 (430--1363)	0.367	1910^bc^ (1045--2635)	0.470
		CT	2182 (1187--2826)		800 (370--4265)		1605 (488--3013)	
	Q	TT	4823		2895 (830--4960)		3570	

Data are expressed as the median and interquartile range (IQR; 25% to 75%). ^a ^= *P*-value based on the Kruskal--Wallis or Mann--Whitney U test. Statistical differences in the mannose-binding lectin (MBL) levels were analyzed according to genotype within each subgroup. ^b ^= *P *< 0.05 vs. the control group. ^c ^= *P *< 0.05 vs. the severe sepsis group. SNP = single nucleotide polymorphism.

**Figure 1:**
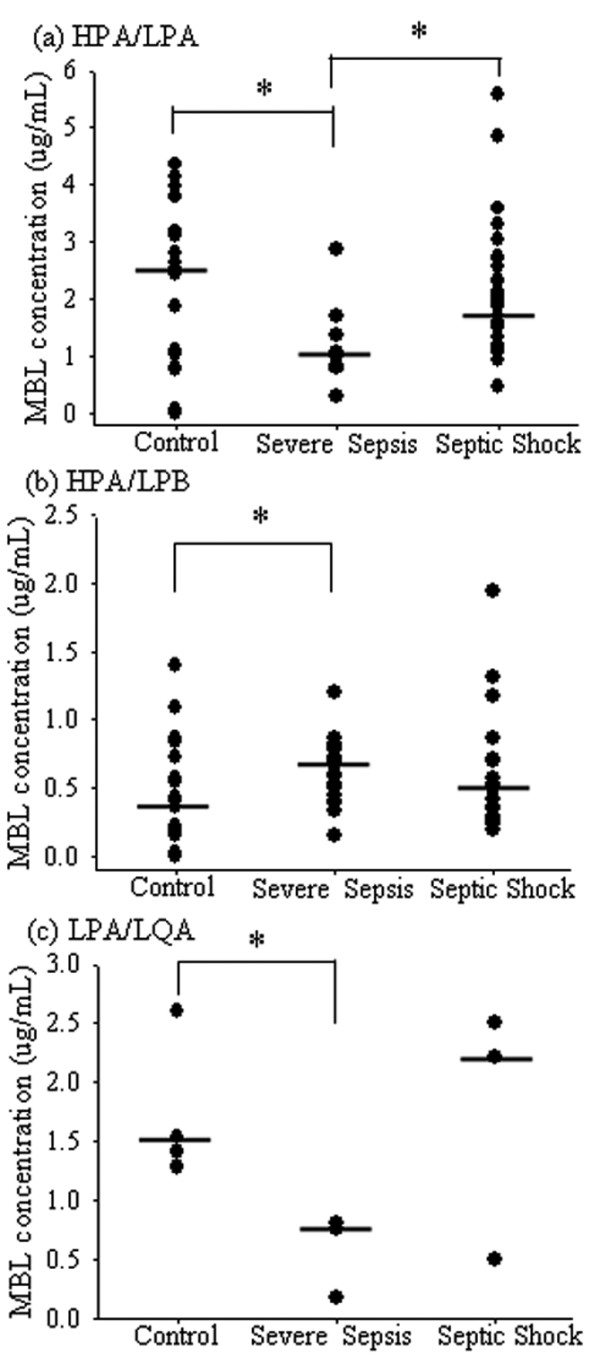
Comparison of serum MBL levels in various haplotypes in the three groups. The serum MBL level in the severe sepsis group was lower than the control group or the septic shock group (*P *< 0.05) for subjects with the *HPA/LPA *haplotype (a). The serum MBL level in the control group was lower than the severe sepsis group (*P *< 0.05) for subjects with the *HPA/LPB *haplotype (b) and higher than the severe sepsis group for subjects with the *LPA/LQA *haplotypes (c). Haplotype estimation was performed using Arlequin software. The median mannose-binding lectin (MBL) levels are indicated by horizontal bars. * *P *< 0.05 between the two groups.

We next analyzed the haplotype profiles to characterize the combined effects of the three polymorphisms. *HPA/HPA*, *HPA/LPA*, and *HPA/LQA *were high MBL-producing haplotypes, and their frequencies were similar among the three groups. For subjects with the *HPA/LPA *haplotype, the serum MBL level was higher for those in the septic shock group than for those in the severe sepsis group (*P *< 0.05). The serum MBL level in the control group was higher than the severe sepsis group for subjects with the *HPA/LPA *or *LPA/LQA *haplotypes (*P *< 0.05) and lower than the severe sepsis group for subjects with the *HPA/LPB *haplotype (*P *< 0.05) (Figure 1).

### The association of serum MBL levels with outcome

**Figure 2:**
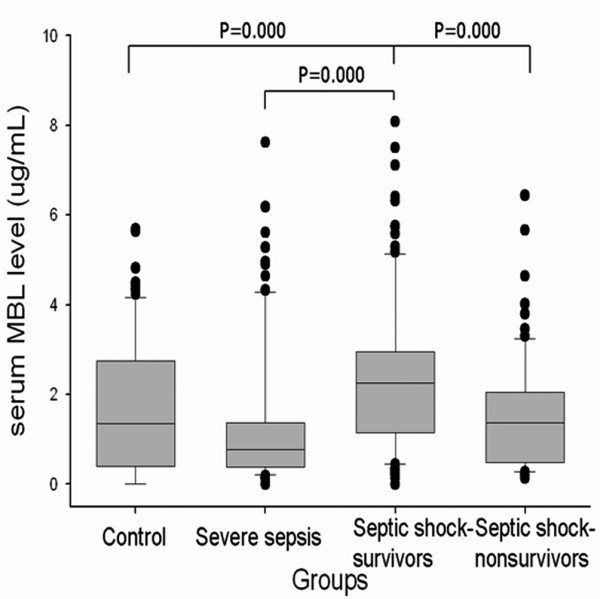
The serum MBL levels in the control group and in the patients with severe sepsis and septic shock. The serum mannose-binding lectin (MBL) level in the septic shock group was higher than the severe sepsis or control group. The survivors had a higher serum MBL level than the nonsurvivors in the septic shock group. Data are expressed as the median and interquartile range (IQR). The box represents the median and IQR (25% to 75%) and the error bar represents the IQR (10% to 90%).

**Figure 3:**
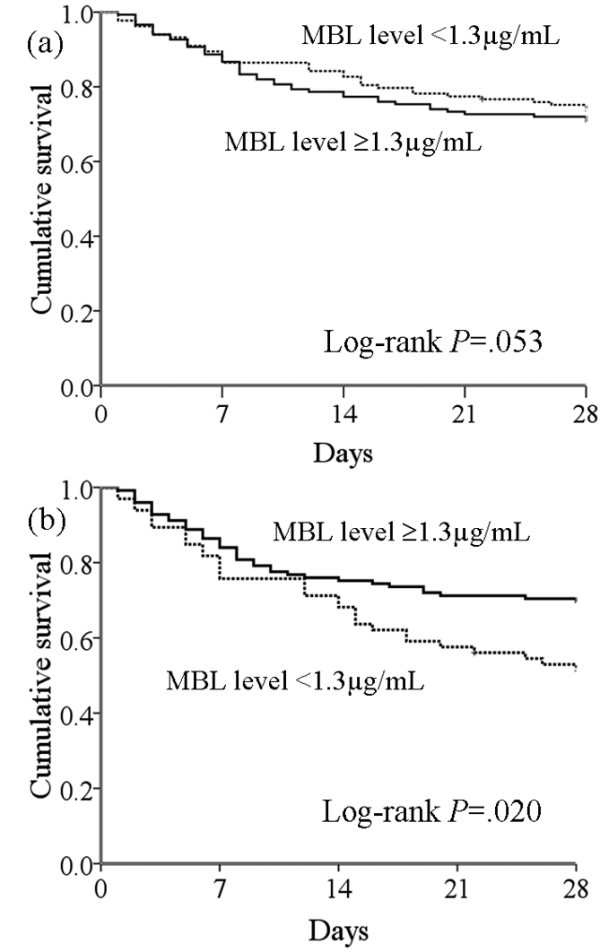
Kaplan-Meier survival curve for septic patients at 28 days according to the MBL level. (a) The difference for 28-day mortality was not found in all patients according to serum MBL levels (MBL <1.3 μg/mL and MBL ≥ 1.3 μg/mL). (b) In subgroup analysis, the difference for 28-day mortality within septic shock patients was more pronounced (*P *= 0.020). A low MBL level (<1.3 μg/mL) was an independent risk factor for mortality after 28 days within the septic shock group. There was a hazard ratio of 0.571 (95% confidence interval, 0.355 to 0.916; *P *= 0.020) in the Cox proportional hazards model correcting for age, sex, and comorbidities. MBL = mannose-binding lectin.

The serum MBL level in the septic shock group (1.85 μg/mL; IQR, 0.87 to 2.67) was higher (*P *< 0.05) than the severe sepsis (0.78 μg/mL; IQR, 0.39 to 1.37) or control groups (1.36 μg/mL; IQR, 0.39 to 2.74); however, the serum MBL level was not significantly different between the severe sepsis and control groups. Subgroup analysis of the septic shock group indicated that the survivors in this group (2.18 μg/mL; IQR, 1.15 to 2.95) had a higher serum MBL level (*P *< 0.05) than the non-survivors (1.37 μg/mL; IQR, 0.49 to 2.05) (Figure 2). We divided the septic patients into two groups according to serum MBL levels (MBL <1.3 μg/mL and MBL ≥ 1.3 μg/mL) using a ROC curve. There was no difference in frequency for Gram-positive or Gram-negative infection depending on the MBL deficiency. According to the Cox proportional hazards model, a low MBL level (<1.3 μg/mL) was an independent risk factor for mortality after 28 days within the septic shock group (Figure 3).

## Discussion

Our study shows that two polymorphisms in *MBL2 *(at codons 54 in exon 1 and -550 in the promoter) may be associated with the severity of sepsis in Korean patients; however, these polymorphisms were not associated with mortality. The serum MBL level was associated with increased risk for mortality after 28 days in the patients with septic shock as found in previous studies [21,27]. However, the serum MBL level was not determined through the known polymorphisms of MBL in the septic condition.

MBL deficiency has been associated with infections in infants and in patients with concomitant immunodeficiencies [15,28,29]. Recent studies have reported that the frequency of MBL-variant alleles is increased with the severity of sepsis [21,27,30]. The functionality of the MBL-2 exon 1 and promoter polymorphisms at -221 G/C, termed *Y/X*, has been well documented in Caucasian patients [22,31,32]. To examine the importance of MBL-variant alleles in the susceptibility to sepsis among Korean patients, we analyzed three polymorphisms (-550, Gly54Asp, and +4) that had previously exhibited significant correlations with the serum MBL level [23].

In the present study, the genotypes of individual SNPs were not independently associated with the development of sepsis. However, homozygosity for the *MBL2 *structural genotype (*A/A*) and the -550 genotype (*H/H*) was associated with the progression from severe sepsis to septic shock. Due to selective pressure promoting heterozygosity, a heterozygous advantage (heterosis) of the MBL2-variant alleles has been proposed [19,33]. The high frequency of MBL-variant alleles in different populations indicates that these polymorphisms represent a balanced genetic system favoring variant alleles arising from genetic selection. Thus, the normal A allele may confer disadvantages to the host under some circumstances, such as sepsis [34]. Although heterozygosity associated with a low MBL level showed an advantage for severity in the sepsis, there was no demonstrable influence on outcome. Accordingly, MBL polymorphisms may play a key role in the severity of sepsis, but they are not a determinant of the outcome.

In contrast, the serum MBL level in response to sepsis seems to be related to the outcome. A MBL level of 1.3 μg/mL or more was an independent factor in the survival of septic shock. However, among patients with the same haplotypes, the serum MBL level was different depending on the clinical setting. This suggests that other factors, such as cytokine levels or other alleles moving in tight linkage disequilibrium, may affect the level of MBL during sepsis. These findings may help explain why, despite the strong relation between *MBL2 *genetic variants and susceptibility to septic shock, there is no evidence to date showing the influence of the *MBL2 *genotype on clinical outcome.

Using *MBL2 *genotype analysis, several studies have shown variations in ethnic-specific genetic structure as well as non-genetic factors [18-20,27]. However, the observation that a deficiency in the amount of functional MBL increases the severity of sepsis has been made repeatedly [27,30]. Therefore, measuring the serum MBL level may be important for the prognosis of septic patients in a clinical setting.

There were certain limitations to the present study. It has been reported that two promoter polymorphisms -550 and -221, and coding variants at codon 52, 54, and 57 of the MBL gene affect the MBL protein level in various populations. In more than 100 Korean controls, the codon 52 and 57 polymorphisms were not present and the effect of the -550 promoter SNP on MBL levels was stronger than that of the -221 promoter SNP. The effect of the X allele at -221 did not reach statistical significance (*P *= 0.156) in the correlation between MBL genotypes and MBL serum levels [23]. This could be because of the low frequency of the X allele in Koreans, 0.11, compared with 0.195 in the Danish population. As we screened only three known polymorphisms based on these results, we cannot rule out the possibility that the observed differences in MBL concentration are at least partially under the influence of additional polymorphisms. In addition, without examining family samples for inheritance patterns, the accuracy of this method of inference is unknown.

Another limitation of our study was that we did not evaluate patients without sepsis, such as patients with noninfectious systemic inflammatory response syndrome. If we had included patients with noninfectious systemic inflammatory response syndrome, we may have been able to explain our results more clearly and provide more support for the suggestion that there is an association of the homozygous *MBL2 *structural genotype (*A/A*) and the -550 genotype (*H/H*) with the progression from severe sepsis to septic shock. However, there were very few patients without infection or with sepsis without organ failure admitted to the medical ICU of the tertiary referral hospital.

Moreover, we measured MBL levels only once within the initial 24 hours of the septic course. This single measurement may reduce the power of the MBL level in terms of a prognostic factor. In addition, certain confounding factors, such as treatment and duration of illness before admission to the ICU, were not included in our analysis.

## Conclusions

Our results showed that the genotype and serum level for *MBL2 *may have different clinical implications, and suggest that the patient with high *MBL2 *production responding to a bacterial invasion may have better prognosis irrespective of *MBL2 *gene polymorphism.

## Key messages

• Homozygosity for the *MBL2 *structural genotype (*A/A*) and the -550 genotype (*H/H*) was associated with the progression from severe sepsis to septic shock.

• An MBL level of 1.3 μg/mL or more showed significantly lower 28-day mortality (*P *= 0.020; hazard ratio, 0.571; 95% CI, 0.355 to 0.916) in the septic shock group.

• The genotype and serum level for *MBL2 *may have different clinical implications.

## Abbreviations

APACHE: Acute Physiology and Chronic Health Evaluation; bp: base pair; CI: confidence interval; ELISA: enzyme-linked immunosorbent assay; ICU: intensive care unit; IQR: interquartile range; MASP: MBL-associated serine proteases; MBL: mannose-binding lectin; M:F: male;female; OR: odds ratio; PCR: polymerase chain reaction; ROC: receiver operator characteristic; SNP: single nucleotide polymorphism; SOFA: Sequential Organ Failure Assessment; TNF: tumor necrosis factor.

## Competing interests

The authors declare that they have no competing interests.

## Authors' contributions

HJW and KYS initiated the study. LCM and HSB participated in patient management. HJW, SKY and YJS analyzed the data. All the authors contributed to read and approved the final manuscript.

## Supplementary Material

Additional file 1 Word file containing a table that lists the Criteria of sepsis, severe sepsis, and septic shock used in our research. Click here for file

Additional file 2 Word file containing a table that lists the Hardy-Weinberg-test for the study population (healthy controls and septic patients). Click here for file
