# Supplementary material for: Association of mannose-binding lectin-2 genotype and serum levels with prognosis of sepsis
Source: Crit Care. 2009 Nov 5;13(6):R176. doi: 10.1186/cc8157 (PMC2811900; doi:10.1186/cc8157)
Supplement: Additional file 1 — Word file containing a table that lists the Criteria of sepsis, severe sepsis, and septic shock used in our research. [file cc8157-S1.DOC]

Supplement 1. Criteria of sepsis, severe sepsis, and septic shock

| Condition | Criteria |
| --- | --- |
| Sepsis | Systemic response to infection, manifested by two or more of the following conditions as a result of infection: a) temperature >38°C or <36°C; b) heart rate >90 beats/min; c) respiratory rate >20 breaths/min or PaCO_2_ <32 mm Hg; and d) white blood cell count >12,000/mm^3^, <4,000/mm^3^, or >10% immature forms. |
| Severe sepsis | Sepsis associated with organ dysfunction, hypoperfusion (serum lactic acid >4 mmol/L), or hypotension (mean arterial pressure <65 mm Hg) |
|  | If hypotension was restored with fluid resuscitation and short term of dopamine within the first six hours. |
| Septic shock | Sepsis with arterial hypotension, despite adequate fluid resuscitation.  If vasopressors (dopamine and norepinephrine) were continued to maintain the blood pressure. |
