# Supplementary material for: Association of mannose-binding lectin-2 genotype and serum levels with prognosis of sepsis
Source: Crit Care. 2009 Nov 5;13(6):R176. doi: 10.1186/cc8157 (PMC2811900; doi:10.1186/cc8157)
Supplement: Additional file 2 — Word file containing a table that lists the Hardy-Weinberg-test for the study population (healthy controls and septic patients). [file cc8157-S2.DOC]

Supplement 2. HW-test for the study population (healthy controls and septic patients)

|  | Study population |
| --- | --- |
|  | *P* value |
| –550, G/C | 0.15 |
| 5′–UTR, +4, C/T | 0.12 |
| Gly54Asp, exon 1 | 0.17 |

The genotype frequencies were checked for consistency among cases (severe sepsis group and septic shock group) and controls separately with those expected from the Hardy–Weinberg equilibrium
